# Electrochemical Migration Study on Sn-58Bi Lead-Free Solder Alloy Under Dust Contamination

**DOI:** 10.3390/ma17215172

**Published:** 2024-10-24

**Authors:** Fuye Lu, Han Sun, Wenlong Yang, Tianshuo Zhou, Yunpeng Wang, Haoran Ma, Haitao Ma, Jun Chen

**Affiliations:** 1 School of Materials Science and Engineering, Dalian University of Technology, Dalian 116000, China; fuyel@mail.dlut.edu.cn (F.L.); 12405053@mail.dlut.edu.cn (H.S.); ywl@mail.dlut.edu.cn (W.Y.); zhou22205039@mail.dlut.edu.cn (T.Z.); yunpengw@dlut.edu.cn (Y.W.); 2 School of Microelectronics, Dalian University of Technology, Dalian 116024, China; mhr@dlut.edu.cn

**Keywords:** Sn-58Bi solder, corrosion, electrochemical migration, dendrite

## Abstract

With the development of electronic packaging technology toward miniaturization, integration, and high reliability, the diameter and pitch of solder joints continue to shrink. Adjacent solder joints are highly susceptible to electrochemical migration (ECM) due to the synergistic effects of high-density electric fields, water vapor, and contaminants. Dust has become one of the non-negligible causal factors in ECM studies due to air pollution. In this study, 0.2 mM/L NaCl and Na_2_SO_4_ solutions were used to simulate soluble salt in dust, and the failure mechanism of an Sn-58Bi solder ECM in the soluble salt in dust was analyzed by a water-droplet experimental method. It was shown that the mean failure time of the ECM of an Sn-58Bi solder in an NaCl solution (53 s) was longer than that in an Na_2_SO_4_ solution (32 s) due to the difference in the anodic dissolution characteristics in the two soluble salt solutions. XPS analysis revealed that the dendrites produced by the ECM process were mainly composed of Sn, SnO, and SnO_2_, and there were precipitation products—Sn(OH)_2_ and Na_2_SO_4_—attached to the dendrites. The corrosion potential in the NaCl solution (−0.351 V) was higher than that in the Na_2_SO_4_ solution (−0.360 V), as shown by a polarization test, indicating that the Sn-58Bi solder had better corrosion resistance in the NaCl solution. Therefore, an Sn-58Bi solder has better resistance to electrochemical migration in an NaCl solution compared to an Na_2_SO_4_ solution.

## 1. Introduction

The interconnecting materials used in microelectronic soldering technology are the material basis for realizing a reliable connection of solder joints. In order to meet the demand for good micro-soldering, lead-free solders such as Sn-Ag, Sn-Zn, Sn-Cu, Sn-Bi, etc. have become hot spots in the research field of electronic packaging [1,2]. Among the aforementioned alloys, the eutectic Sn-58Bi solder alloy (Sn-58 wt.% Bi) is distinguished by its low melting point, and it can meet the requirements of low temperature soldering and is widely used in the assembly of devices with low heat resistance, such as microelectronic sensors and flexible circuit boards. In addition, it has good wettability and high tensile strength, providing it with wide application prospects in the field of low-temperature soldering [3,4,5].

In recent years, electronic devices have evolved towards miniaturization, high integration, and multifunctionality. The surface density of a printed circuit board has increased and the size and spacing of the solder balls/bonding wires have decreased, resulting in a high susceptibility to inter-wire insulation failures caused by ECM [6,7,8,9]. ECM is a special form of corrosion. It is the formation of metal ions due to dissolution at one electrode. These metal ions move through the electrolyte to another electrode and undergo reductive deposition to form metal deposits [10]. The shape of the sediment is dendritic, and it is generally referred to as a “dendrite” [11]. As the dendrite grows from the cathode to the anode, the surface insulation resistance between the two poles will decrease significantly and a short-circuit failure will occur [12,13]. Reduced insulation resistance can lead to heat buildup between conductive paths, reducing the reliability of electronic circuits and electrical equipment [14,15,16].

In an actual working environment, natural dust is prevalent, and it can get inside electronic equipment, affecting the performance of circuit boards and even causing insulation failures between neighboring solder joints. Therefore, it is essential to study the effect of dust contaminants on the ECMs of solders [17,18,19]. Soluble salts in natural dust include anions such as F^−^, Cl^−^, NO^3−^, and SO_4_^2−^ and cations such as Na^+^, NH^4+^, K^+^, Mg^2+^, and Ca^2+^ [17]. In ECM studies, NaCl and Na_2_SO_4_ are commonly used to simulate soluble salts in dust. Also, NaCl and Na_2_SO_4_ have the same cation, which makes it easier to compare the effect of anions on the electrochemical migration of a solder.

According to Bo Song et al. [17], it took approximately 9 months for dust deposition to reach a distribution density of 150 μg/cm^2^ indoors. Its solution conductivity was comparable to that of a 2.2 mM/L NaCl solution. Since natural dust is a multi-layered structure of soluble salts wrapped around insoluble particles, the soluble salts cannot be completely dissolved in a water film condensed on the surface of a circuit board at a certain humidity, and the density of dust deposits inside electronic devices is usually lower than the density of dust deposits in indoor environments. Therefore, a low concentration of a 0.2 mM/L NaCl and Na_2_SO_4_ solution was selected in this study to simulate the effect of soluble salts in dust on the ECM of an Sn-58Bi solder. The ECM phenomenon on the Sn-58Bi solder was observed in situ using a water-drop experimental method, and the corrosion resistance of the solder in the NaCl and Na_2_SO_4_ solutions was investigated by testing the polarization curves to determine its ECM resistance.

## 2. Materials and Methods

### 2.1. Sample Preparation

**Figure 1:**
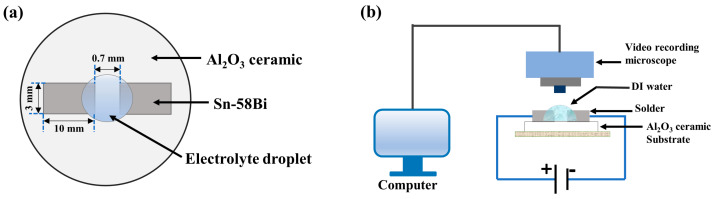
(**a**) Top view of the electrode, and (**b**) schematic of the water droplet experimental setup.

Eutectic Sn-58Bi solder sheets with dimensions of 10 × 3 × 0.2 mm were selected. First, a cross section of the Sn-58Bi solder was progressively ground with 400–800–1200–1500–2000 mesh silicon carbide water-abrasive sandpaper to expose the solder matrix. After sanding to ensure that the scratches were uniform and of equal depth, the solder was rinsed with anhydrous ethanol and distilled water and blown dry with cold air. Then, the Sn-58Bi solder was fixed as an electrode on a ceramic substrate with a distance of 0.7 mm between the two electrodes, as shown in Figure 1a.

### 2.2. Experimental System Construction

The water droplet experimental setup is shown in Figure 1b. The prepared samples were placed under a stereomicroscope (Olympus SZX12, Tokyo, Japan), and each end of the sample was connected to an electrode of the electrochemical workstation. NaCl and Na_2_SO_4_ solutions with concentrations of 0.2 mM/L were used as the electrolytes. The electrolyte content was 5 μL. The bias voltage was 3 V. An electrochemical workstation was set up to measure and record the change in current through the electrode and between the electrolytes over time. At the same time, the formation of dendrites was observed under a stereomicroscope, and the whole process was recorded by a video camera. To ensure the reproducibility of the results, seven parallel experiments were performed in the NaCl and Na_2_SO_4_ solutions.

To measure the pH change near the electrode during the ECM, a few drops of liquid universal pH reagent (pH = 1–14) were added to a 0.2 mM/L solution of NaCl and Na_2_SO_4_. The color was medium yellow, indicating that the solution was neutral. The color change of the electrolyte during the ECM process was observed by stereomicroscopy to determine the alkalinity, which was used to analyze the growth mechanism of the dendrites.

### 2.3. Polarization Test

To investigate the effect of the NaCl and Na_2_SO_4_ solutions on the corrosion resistance of the Sn-58Bi solder, the polarization curves of the solder were measured using the dynamic potential scanning method. An electrochemical workstation (CS350M) and a three-electrode system were used for the testing. The solder was used as the anode, the Pt auxiliary electrode was used as the cathode, and the saturated calomel electrode (SCE) was used as the reference electrode. Measurements were performed in the 0.2 mM/L NaCl and Na_2_SO_4_ solutions at room temperature. The potential scanning range was −0.5 to 1.5 V, the test potential was scanned from negative to positive, and the scanning rate was 1 mV/s. Before the polarization curve test, an open circuit potential (OCP) was measured. To ensure the reproducibility of the results, three separate parallel experiments were performed.

### 2.4. Ex Situ Characterizations

After the ECM experiment, the samples were allowed to dry naturally at room temperature. Then, the samples were ion-sputtered with gold to make them conductive, and the morphology of the electrochemical migration products was characterized by scanning electron microscopy (SEM, Zeiss supra 55 (Vp), Oberkochen, Germany). Corrosion product microstructures and chemical elements after the polarization test were analyzed through an electron probe X-ray microanalyzer (EPMA, JXA-8530F PLUS, Japan Electron Optics Laboratory Co., Ltd., Tokyo, Japan). An XPS (ESCALAB 250Xi, Waltham, MA, USA) analysis of the dendrite was performed. The base pressure in the experimental chamber was in the range of 10^−10^ mbar. The spectra were measured with Al Ka (hv = 1486.6 eV) radiation, and the overall energy resolution was approximately 0.45 eV.

## 3. Results

### 3.1. Effect of the NaCl and Na_2_SO_4_ Solutions on the ECM of the Sn-58Bi Solder

**Figure 2:**
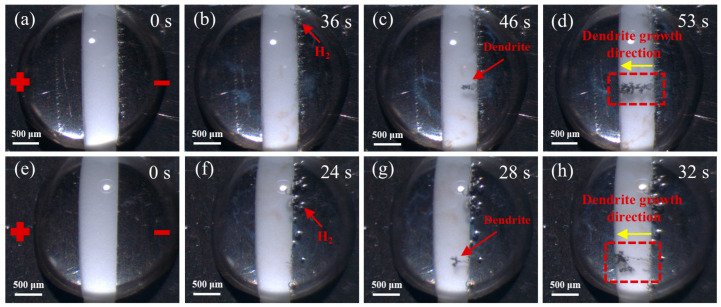
In situ observation of the electrochemical migration phenomena of the Sn-58Bi solder in the 0.2 mM/L NaCl and Na_2_SO_4_ solutions ((**a**–**d**), NaCl solution; (**e**–**h**), Na_2_SO_4_ solution; anode on the left, cathode on the right).

Figure 2 shows the in situ observation of the ECM phenomenon in an Sn-58Bi solder in the 0.2 mM/L NaCl and Na_2_SO_4_ solutions. From Figure 2a, it can be seen that in the NaCl solution, a small number of gas bubbles were produced at the cathode after a period of energization (Figure 2b), and this was due to the production of hydrogen (H_2_) from the water reduction. With the extension of time, the cathode began to form dendrites accompanied by precipitation, and after 46 s, dendrite formation could be clearly observed (Figure 2c), indicating that a long period of time had been experienced after energization before the dendrite formation was observed at the cathode. Once the branch crystals grew, they quickly grew to the anode. After 53 s, the dendrites grew from the cathode to the anode, resulting in a short circuit between the cathode and the anode bridges (Figure 2d). Thus, the ECM short-circuit failure time of the Sn-58Bi solder in the NaCl solution was 53 s. In the Na_2_SO_4_ solution, a large number of gas bubbles were produced at the cathode shortly after energization compared to the NaCl solution, indicating that a violent reduction reaction occurred at the cathode in the Na_2_SO_4_ solution (Figure 2f). After 28 s, dendrites appeared at the cathode and a large amount of precipitate was produced (Figure 2g). After 32 s, the dendrite reached the anode and a short circuit occurred (Figure 2h). It can be seen that the electrochemical migration short-circuit failure time of the Sn-58Bi solder in the Na_2_SO_4_ solution was 32 s.

**Figure 3:**
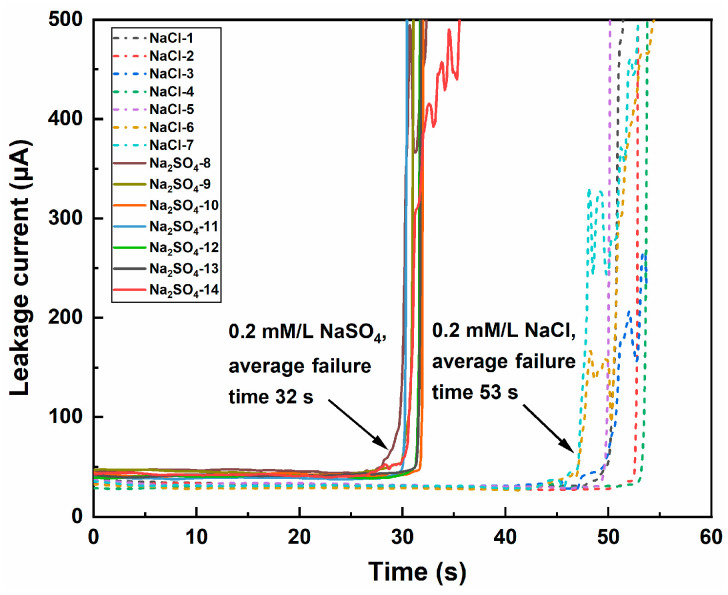
Current–time curves of the Sn-58Bi solder for the ECM tests (1–7, 0.2 mM/L NaCl solution; 8–14, 0.2 mM/L Na_2_SO_4_ solution).

Figure 3 shows the current–time curves of the Sn-58Bi solder for the ECM tests 1–7 in the 0.2 mM/L NaCl solution and 8–14 in the 0.2 mM/L Na_2_SO_4_ solution. The figure shows that the current increased dramatically after the power had been turned on for some time, and this was due to the growth of the dendrites from the cathode to the anode. The mean failure time of the Sn-58Bi solder in the Na_2_SO_4_ solution (32 s) was shorter than that of the NaCl solution (53 s). This was due to the different anodic dissolution characteristics of the different solutions. The faster the anodic dissolution of the metal, the shorter the failure time, and the magnitude of the electrochemical migration susceptibility corresponded well to the magnitude of the solder corrosion resistance in the solution [20].

### 3.2. Inter-Electrode pH Distribution During the ECM Process

**Figure 4:**
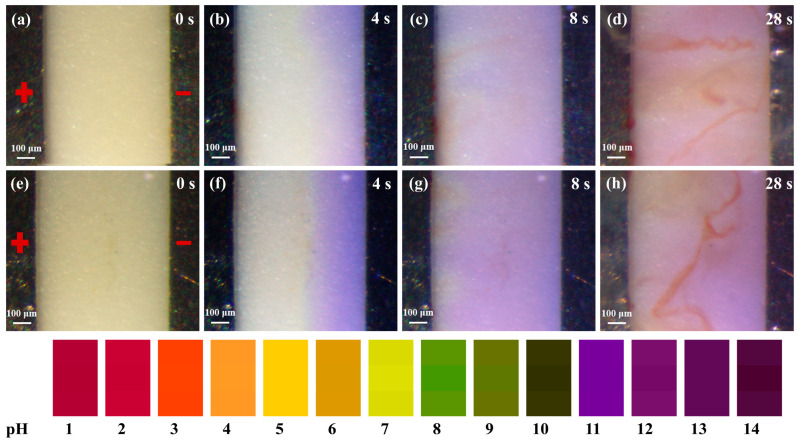
In situ observation of the pH distribution between the electrodes in the 0.2 mM/L NaCl and Na_2_SO_4_ solutions ((**a**–**d**), NaCl solution; (**e**–**h**), Na_2_SO_4_ solution; anode on the left, cathode on the right).

In order to understand the pH distribution between the two electrodes during the ECM test, a few drops of liquid pH reagent were added to a 0.2 mM/L solution of NaCl and Na_2_SO_4_ to observe the pH change between the electrodes. As shown in Figure 4a,e, in the initial stage, the electrolyte droplets were light yellow in color and had a pH of between 6 and 8. After applying the bias voltage, a purple color was observed near the cathode. This indicated that the OH^−^ produced by the cathodic reduction reaction increased the alkalinity of the droplet, while the acidity of the droplet increased at the anode (Figure 4b,f). As the excitation time was further increased, the OH^−^ concentration at the cathode increased due to the bubble evacuation convection effect at the cathode surface [21]. This allowed more OH^−^ to migrate quickly toward the anode, resulting in an increasingly larger purple region (Figure 4c,g). The anodically dissolved Sn^2+^ migrated to the cathode at a relatively slow rate, causing the OH^−^ and Sn^2+^ to precipitate on the anodic side of the bias. Eventually, due to the convection effect, the resulting precipitate moved toward the cathode and was distributed between the cathode and anode, as shown in Figure 4d,h.

### 3.3. Microscopic Morphology and Compositional Analysis of the Migration Products

**Figure 5:**
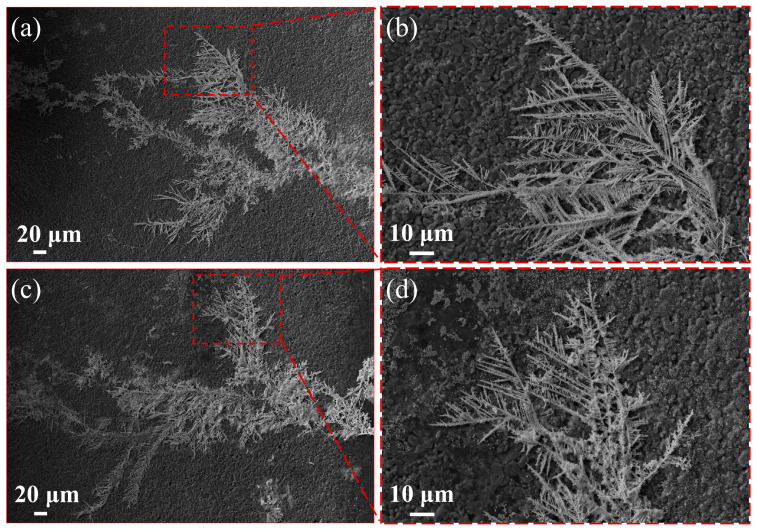
SEM images of the dendrites formed by the electrochemical migration process of the Sn-58Bi in the 0.2 mM/L NaCl and Na_2_SO_4_ solutions ((**a**,**b**), NaCl solution; (**c**,**d**), Na_2_SO_4_ solution; anode on the left, cathode on the right).

During the ECM process of the Sn-58Bi solder, metal ions generated by the anodic dissolution migrated toward the cathode, where they were reduced to dendrites and grew toward the anode. SEM and XPS were used to analyze the morphology and composition of the dendrites. Figure 5 shows an SEM image of the Sn-58Bi solder in the 0.2 mM/L NaCl and Na_2_SO_4_ solutions during the ECM process, which produced dendrites. The microscopic morphology of the ECM dendrite products of the Sn-58Bi solder in the NaCl and Na_2_SO_4_ solutions were relatively similar, and both of them were typical tree-like dendrites. The main trunk of the branch crystals was distributed with many fine branches, which were at an angle to the main trunk and showed a clear dendritic shape. This was in agreement with the results of the existing literature [22]. In addition, there was a white precipitate attached to the surface of the dendrite. It is noteworthy that the dendrites produced in the Na_2_SO_4_ solution were finer and dendritic or needle shaped. This was mainly due to the fact that the Sn-58Bi solder dissolved faster in the Na_2_SO_4_ solution. The accelerated anodic dissolution promoted the migration of metal ions, leading to a significant increase in the nucleation and growth of the dendrites at the cathodic edge so that the dendrites exhibited an elongated shape (Figure 5c,d).

**Figure 6:**
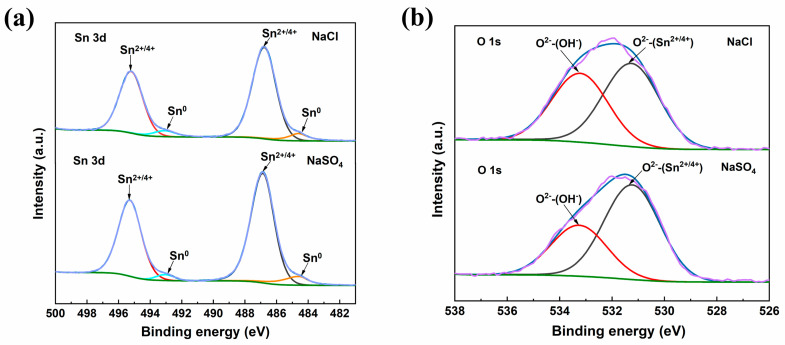
High resolution XPS spectra of the Sn-58Bi in the 0.2 mM/L NaCl and Na_2_SO_4_ solutions for the ECM process’s dendrite products corresponding to Sn 3d and O 1s ((**a**), Sn 3d; (**b**), O 1s).

Figure 6 shows the XPS high-resolution spectra of Sn 3d and O 1s in the dendrites. Obviously, in the Sn 3d spectrum, 484.7 eV and 493.1 eV were the characteristic peaks of the Sn_0_, and 486.8 eV and 495.2 eV correspond to the characteristic peaks of Sn^2+^/Sn^4+^ (Figure 6a). In the O 1s spectra, peaks at 531.3 eV and 533.3 eV corresponding to O^2−^ and OH^−^ were observed (Figure 6b) [23]. Therefore, it could be determined that the dendrites produced by the EMC process in the NaCl and Na_2_SO_4_ solutions contained mainly Sn, SnO, and SnO_2_ [24]. Combined with the observation of the white precipitates attached to the surface of the dendrites in the SEM images, it could be judged that the white precipitates were Sn(OH)_2_ and Sn(OH)_4_. In addition, the XPS peak intensities of the Sn^2+/4+^ and O^2−^ for the dendrites produced in the Na_2_SO_4_ solution were significantly higher than those of the NaCl solution, indicating that the Sn-58Bi solder had more dissolved Sn content in the Na_2_SO_4_ solution and the dendrites that formed also had higher Sn contents. In contrast, in the O 1s spectra, the OH^−^ peak area of the dendrimer product in the NaCl solution was higher than that in the Na_2_SO_4_ solution, indicating that more Sn(OH)_2_ and Sn(OH)_4_ precipitates were formed in the NaCl solution. The resulting precipitate acted as a spatial barrier, preventing anodic dissolution and ionic migration and allowing the growth of the dendrites to be inhibited [25].

## 4. Discussion

### 4.1. Electrochemical Reactions During the ECM of the Sn-58Bi Solder in the NaCl and Na_2_SO_4_ Solutions

The ECM process includes several processes such as the dissolution of metal at the anode, diffusive migration of metal ions to the cathode, and reductive aggregation of metal ions at the cathode. The products of the ECM process are dendrites and precipitates. The standard electrode potentials for Sn and Bi in the Sn-58Bi solder were E_Sn_ = −0.138 V (SHE) and E_Bi_ = +0.317 V (SHE), respectively [22]. Due to the large difference between the standard electrode potentials of the Sn and Bi, during the ECM process, only the Sn participates appeared in the anodic dissolution reaction. The probability of oxidation was inversely proportional to the standard electrode potential value, and so the Bi in the Sn-58Bi solder did not precipitate in the dissolution of the anode [26]. The chemical reactions that occur during ECM are as follows: 

Anodic:

(1) $$\mathrm{Sn}-\;{2e}^{-}\to {\mathrm{Sn}}^{2+},$$

(2) $${\mathrm{Sn}}^{2+}-{2e}^{-}\to {\mathrm{Sn}}^{4+},\;\mathrm{and}$$

(3) $${2H}_{2}O\;-\;{4e}^{-}\to {4H}^{+}+O_{2}.$$

Cathodes:

(4) $${2H}_{2}O\;+\;{2e}^{-}\to {2\mathrm{OH}}^{-}+H_{2}\;,$$

(5) $${2H}_{2}O\;+\;O_{2}+{4e}^{-}\to {4\mathrm{OH}}^{-},$$

(6) $${\mathrm{Sn}}^{2+}+{2e}^{-}\to \mathrm{Sn},\;\mathrm{and}$$

(7) $${\mathrm{Sn}}^{4+}+{4e}^{-}\to \mathrm{Sn}.$$

Precipitates:

(8) $${\mathrm{Sn}}^{2+}+{2\mathrm{OH}}^{-}\to {\mathrm{Sn}(\mathrm{OH})}_{2},$$

(9) $${\mathrm{Sn}}^{4+}+{4\mathrm{OH}}^{-}\to {\mathrm{Sn}(\mathrm{OH})}_{4},$$

(10) $${\mathrm{Sn}}^{4+}+{4H}_{2}O\to {\mathrm{Sn}(\mathrm{OH})}_{4}+{4H}^{+},\;\mathrm{and}$$

(11) $$\mathrm{Sn}\;+\;{4H}_{2}O\to {\mathrm{Sn}(\mathrm{OH})}_{4}+{4H}^{+}+{4e}^{-}.$$

According to the literature [14], most of the Sn^2+^ generated by anodic dissolution will be further oxidized to Sn^4+^ (Reaction (2)). The dissolution of Sn and the oxidation of water in Sn-58Bi are the main anodic reactions (Reactions (1)–(3)) [27,28]. Oxidation by water promotes the dissolution of some of the metal atoms in the alloy [29]. The main cathodic reactions of the process are the reduction of H_2_O (Reaction (4)), dissolved oxygen (Reaction (5)), and the reduction of Sn^2+/4+^ to metallic tin [30]. The growth of dendrites can be attributed to the direct reduction of Sn^4+^ [25].

During the ECM process, a portion of Sn^2+^/Sn^4+^ migrating from the anode to the cathode encounters OH^−^ migrating from the cathode to the anode, resulting in the formation of the precipitates Sn(OH)_2_ and Sn(OH)_4_. Sn(OH)_2_ is predominantly basic, while Sn(OH)_4_ is predominantly acidic. At room temperature, the solubility products of Sn(OH)_2_ and Sn(OH)_4_ are K_sp_ (Sn(OH)_2_) = 5.45 × 10^−28^ and K_sp_ (Sn(OH)_4_) = 10^−56^, respectively. This means that both substances form precipitates very easily at room temperature [31]. The simultaneous hydrolysis of Sn^4+^ and direct oxidation of Sn also promotes the generation of Sn(OH)_4_ (Reactions (10) and (11)).

As shown in Reactions (11) and (13), Sn(OH)_2_ and Sn(OH)_4_ undergo dehydration upon natural drying in air and form oxides of the SnO and SnO_2_ classes [9]. XPS also confirmed that the dendrites produced by ECM were mainly composed of Sn, SnO, and SnO_2,_ and there were precipitation products (Sn(OH)_2_ and Sn(OH)_4_) attached to the dendrites.

(12) $${\mathrm{Sn}(\mathrm{OH})}_{2}\to \mathrm{SnO}\;+\;{2H}_{2}O\;\mathrm{and}$$

(13) $${\mathrm{Sn}(\mathrm{OH})}_{4}\to {\mathrm{SnO}}_{2}+{2H}_{2}O.$$

### 4.2. Polarization Curves and Corrosion Products

**Figure 7:**
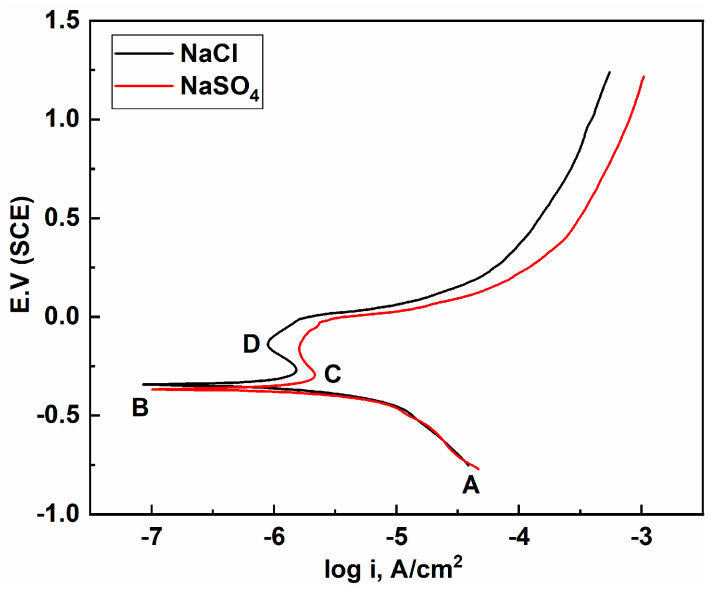
Polarization curves of the Sn-58Bi in the 0.2 mM/L NaCl and Na_2_SO_4_ solutions.

ECM is caused by anodic dissolution. An Sn-58Bi solder has different corrosion potentials in NaCl and Na_2_SO_4_ solutions, and the degree of ECM difficulty is also different. In this study, the corrosion behavior of an Sn-58Bi solder in 0.2 mM/L NaCl and Na_2_SO_4_ solutions was analyzed using polarization curves. As shown in Figure 7, the Sn-58Bi solder exhibited similar polarization behaviors in both solutions. The polarization curves both contained passivation regions (C–D). First, the oxygen dissolution reaction occurred at the interface on the cathodic branch (stage A–B) to generate OH^−^. As the potential increased further in the positive direction to E_corr_, the solder alloy began to undergo anodic polarization at point B. The B–C stage was dominated by anodic polarization and substance dissolution reactions. Subsequently (stage C–D), there was a small decrease in the corrosion current, which was due to the corrosion of the Sn-rich phase on the surface of the alloy and the formation of a passivation film (SnO) on the surface [20]. When the potential exceeded the passivate film breakdown potential (point D), passivity loss occurred and localized corrosion started due to the presence of the Cl^−^ ions, which initiated local corrosion spots that were assumed to be the main reason for the rupture of the passive layer. This was shown as an increase in the current density until the end of the polarization process, indicating the start of the corrosion product formation process [23].

However, there were still differences in the corrosion resistance of the Sn-58Bi solder due to the different corrosion fluids. The corrosion potential (E_corr_) and corrosion current density (I_corr_) were determined by Tafel extrapolation. Table 1 shows the average values of E_corr_ and I_corr_ for the Sn-58Bi solder in the NaCl and Na_2_SO_4_ solutions. It can be seen that the E_corr_ of the Sn-58Bi solder in the Na_2_SO_4_ solution (−0.360 V) was lower than that in the NaCl solution (−0.351 V), indicating that it had poor corrosion resistance and was susceptible to dissolution, and therefore, it was more prone to ECM.

**Figure 8:**
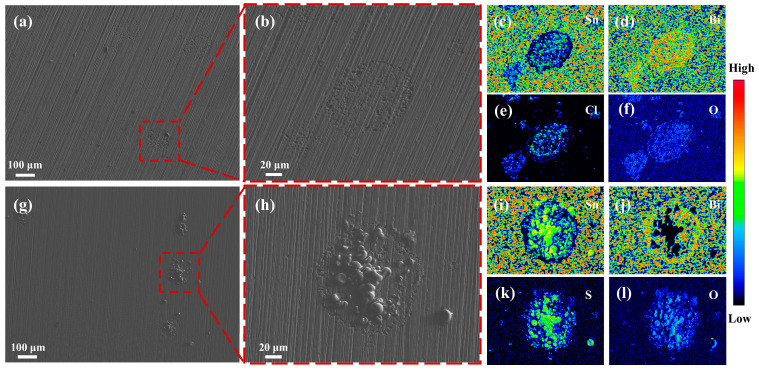
Surface morphology and EDS maps of the sample’s surface after a polarization test on the Sn-58Bi in the 0.2 mM/L NaCl and Na_2_SO_4_ solutions ((**a**–**f**), NaCl solution; (**g**–**l**), Na_2_SO_4_ solution).

Figure 8 shows the surface morphology and EDS maps of the sample after a polarization test on the Sn-58Bi solder in the 0.2 mM/L NaCl and Na_2_SO_4_ solutions. As illustrated in Figure 8a,g, after the polarization test, corrosion pits appeared on the surface of the Sn-58Bi solder, and the corrosion products showed porous structures. The degree of corrosion that occurred in the Na_2_SO_4_ solution was more obvious, indicating that the Sn-58Bi solder was easier to corrode in the Na_2_SO_4_ solution. The EDS surface-scanning map shows that the corrosion products in the Na_2_SO_4_ solution were mainly composed of Sn, S, and O elements, and the corrosion products on the surface of the sample in the NaCl solution were mainly composed of Sn, Cl, and O elements. The Sn bonded with the Cl^−^ to form SnCl_2_, SnCl_4_, and Sn_3_O(OH)_2_Cl_2_ [9]. The possible reaction equation is as follows:

(14) $$3\mathrm{Sn}+2{\mathrm{Cl}}^{-}+4{\mathrm{OH}}^{-}\to {\mathrm{Sn}}_{3}O{\left(\mathrm{OH}\right)}_{2}{\mathrm{Cl}}_{2}+H_{2}O\;+\;{6e}^{-}\;.$$

## 5. Conclusions

In this study, NaCl and Na_2_SO_4_ were selected as representative components of soluble salts in dusty soil. The effects and mechanisms of their actions on the electrochemical migration failure of an Sn-58Bi solder were investigated using a water-droplet experimental method, and the following conclusions were obtained:

(1) Dendrites were produced during the ECM of the Sn-58Bi solder in both the NaCl and Na_2_SO_4_ solutions at concentrations of 0.2 mM/L, and the mean failure time of the ECM in the NaCl solution (53 s) was longer than that in the Na_2_SO_4_ solution (32 s), which was due to the different anodic dissolution characteristics of the different solutions.
(2) The XPS analysis showed that the dendrites produced by the ECM process were mainly composed of Sn, SnO, and SnO_2_, and there were precipitation products (Sn(OH)_2_ and Sn(OH)_4_) attached to the dendrites. Compared to the NaCl solution, more Sn was dissolved at the anode of the ECM process in the Na_2_SO_4_ solution. The resulting dendrites contained more Sn or Sn oxides. In addition, the ECM process in the NaCl solution produced more Sn(OH)_2_ and Sn(OH)_4_ precipitates. These precipitates acted as spatial barriers, hindering anodic dissolution and ion migration and, thus, preventing the growth of dendrites.
(3) After a polarization curve test, the E_corr_ of the Sn-58Bi solder in the NaCl solution (−0.351 V) was found to be higher than that in the Na_2_SO_4_ solution (−0.360 V). Combined with the micromorphology of the sample’s surface after corrosion, it could be found that the degree of corrosion of the Sn-58Bi solder in the Na_2_SO_4_ solution was more obvious. Therefore, it was shown that the Sn-58Bi solder had better corrosion resistance in the NaCl solution, indicating better resistance to electrochemical migration.

## Figures and Tables

**Table 1:** Corrosion parameters extracted from the potentiodynamic polarization curves of the Sn-58Bi solder alloys in the 0.2 mM/L NaCl and Na_2_SO_4_ solutions.

Solution	OCP (mV. SCE)	E_corr_ (mV)	I_corr_ (μA/cm^2^)
NaCl	−261	−351	0.937
Na_2_SO_4_	−283	−360	0.998

The values are the averages of three parallel measurements.

## Data Availability

The original contributions presented in the study are included in the article, further inquiries can be directed to the corresponding author.
